# Flexible Data Rate Allocation Using Non-Orthogonal Multiple Access (NOMA) in a Mode Division Multiplexing (MDM) Optical Power Splitter for System-on-Chip Networks

**DOI:** 10.3390/s23167259

**Published:** 2023-08-18

**Authors:** Yuan-Zeng Lin, Chi-Wai Chow, Tien-Wei Yu, Yin-He Jian, Tun-Yao Hung, Jian-Wen Chen, Chien-Hung Yeh

**Affiliations:** 1 Department of Photonics & Graduate Institute of Electro-Optical Engineering, College of Electrical and Computer Engineering, National Yang Ming Chiao Tung University, Hsinchu 30010, Taiwan; 2 Department of Photonics & Graduate Institute of Electro-Optical Engineering, College of Electrical and Computer Engineering, National Chiao Tung University, Hsinchu 30010, Taiwan; 3 Department of Photonics, Feng Chia University, Taichung 40724, Taiwan

**Keywords:** silicon photonics (SiPh), optical interconnect, mode division multiplexing (MDM), optical power splitter, orthogonal frequency division multiplexing (OFDM), non-orthogonal multiple access (NOMA), genetic algorithm (GA)

## Abstract

We put forward and demonstrate a silicon photonics (SiPh)-based mode division multiplexed (MDM) optical power splitter that supports transverse-electric (TE) single-mode, dual-mode, and triple-mode (i.e., TE_0_, TE_1_, and TE_2_). An optical power splitter is needed for optical signal distribution and routing in optical interconnects. However, a traditional optical splitter only divides the power of the input optical signal. This means the same data information is received at all the output ports of the optical splitter. The powers at different output ports may change depending on the splitting ratio of the optical splitter. The main contributions of our proposed optical splitter are: (i) Different data information is received at different output ports of the optical splitter via the utilization of NOMA. By adjusting the power ratios of different channels in the digital domain (i.e., via software control) at the Tx, different channel data information can be received at different output ports of the splitter. It can increase the flexibility of optical signal distribution and routing. (ii) Besides, the proposed optical splitter can support the fundamental TE_0_ mode and the higher modes TE_1_, TE_2_, etc. Supporting mode-division multiplexing and multi-mode operation are important for future optical interconnects since the number of port counts is limited by the chip size. This can significantly increase the capacity besides wavelength division multiplexing (WDM) and spatial division multiplexing (SDM). The integrated SiPh MDM optical power splitter consists of a mode up-conversion section implemented by asymmetric directional couplers (ADCs) and a Y-branch structure for MDM power distribution. Here, we also propose and discuss the use of the Genetic algorithm (GA) for the MDM optical power splitter parameter optimization. Finally, to provide adjustable data rates at different output ports after the MDM optical power splitter, non-orthogonal multiple access—orthogonal frequency division multiplexing (NOMA-OFDM) is also employed. Experimental results validate that, in three modes (TE_0_, TE_1_, and TE_2_), user-1 and user-2 achieve data rates of (user-1: greater than 22 Gbit/s; user-2: greater than 12 Gbit/s) and (user-1: greater than 12 Gbit/s; user-2: 24 Gbit/s), respectively, at power-ratio (PR) = 2.0 or 3.0. Each channel meets the hard-decision forward-error-correction (HD-FEC, i.e., BER = 3.8 × 10^−3^) threshold. The proposed method allows flexible data rate allocation for multiple users for optical interconnects and system-on-chip networks.

## 1. Introduction

**Figure 1:**
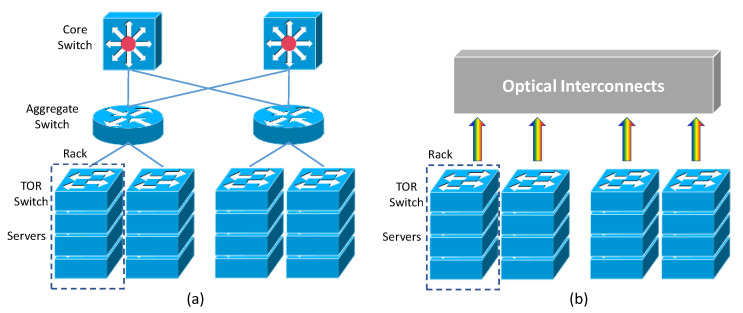
Data center architecture using (**a**) optical point-to-point links and (**b**) all-optical interconnects.

In recent years, the huge increase in Internet bandwidths, driven by broadband services, such as 4K/8K video streaming, cloud computing, social networking, etc., has generated the need for powerful data centers. Inside these data centers, there are thousands of high-performance servers interconnected. The data center performance is highly affected by the performances of these interconnects and switches [1]. Data center networks using electronic switches consume high power and may not satisfy the bandwidth demand required in future data center networks. Optical interconnects have gained much attention recently as a promising solution to provide high bandwidth, high throughput, low latency, as well as low power consumption when compared with electronic switches [2,3,4]. Figure 1a shows the block diagram of a current typical data center architecture [1]. It consists of many servers, and these servers are mounted on racks. When a request is made from a user, a data packet is forwarded via the Internet to the front end of the data center. At the front end, some switches, particularly for content requests, route this request to suitable servers. This request may require synchronization and communication among many servers, such as applications and databases. These servers are mounted in racks and connected via the top-of-rack (TOR) switches [1]. These TOR switches are then interconnected via aggregate and core switches using point-to-point optical links, as shown in Figure 1a. In these point-to-point optical links, optical signals are transmitted between switches and servers via optical fibers. These are similar to the point-to-point optical link in fiber-optics communication. However, these optical signals have to be converted to electrical signals when these point-to-point optical links are terminated at these switches and servers.

In the near future, TOR bandwidth of up to 51.2 Tbit/s is needed [5]. Usually, a data center has more than 10k racks; hence, the total bandwidth requirement is about 512 Pbit/s. To meet this overwhelming bandwidth growth, higher bandwidth links should be adopted. As the electrical switches consume a huge amount of energy for the optical-to-electrical (OE) and electrical-to-optical (EO) conversions for the transceivers (TRxs) and switching in the electronic domain. Novel technology and architecture should enable the data rates inside a data center to grow continuously. Figure 1b illustrates one future data center architecture using all-optical interconnects [1] to eliminate the power-hungry electrical switches and OE and EO transceivers. In these all-optical interconnects [6,7,8,9,10,11,12], optical switching is performed in the optical domain. These could meet the high bandwidth and low power consumption requirements. It is worth noting that optical interconnect can replace the optical switching function and reduce the power consumption during the OE and EO conversions in the electronic domain. However, scheduling and buffering cannot be replaced since the all-optical buffer is immature.

Several optical switches have been proposed. These optical switches are based on micro-electromechanical systems (MEMS) technology [6,7,8], liquid crystals on silicon (LCOS) technology [13,14,15], and semiconductor optical amplifiers (SOA) [16,17,18,19,20]. The selection of an optical switch for the data center application is mainly driven by the cost, power consumption, insertion loss, reconfiguration time, etc. Thanks to the advancements in manufacturing processes of complementary metal-oxide-semiconductor (CMOS), high performance, high yield and low-cost silicon photonics (SiPh) based integrated circuits can be manufactured [21,22,23,24,25,26,27]. Many high-performance SiPh-integrated devices for optical communication have been proposed and demonstrated [28,29,30,31,32]. Besides, using integrated SiPh devices to achieve high-performance optical interconnects and switches has also been realized [33,34,35,36,37]. To further increase the transmission capacity in these optical interconnects, various advanced multiplexing techniques have been utilized to improve spectral efficiency. These multiplexing schemes can be mainly implemented in the physical device domain and digital domain. The multiplexing in the physical domain includes wavelength division multiplexing (WDM) [38], polarization division multiplexing (PolDM) [39], and mode division multiplexing (MDM) [40,41]. Among these, MDM is an effective technique for enhancing the overall transmission capacity in SiPh based interconnects. High-performance MDM mode multiplexers (Mux) and demultiplexers (Demux) can be realized using asymmetric directional couplers (ADCs) [42]. The implementation of MDM Mux and Demux based on ADCs has achieved high-capacity transmission exceeding Tbit/s [43,44,45,46].

Besides the WDM, PolDM and MDM that can be realized in the physical domain via the SiPh chip, digital multiplexing technologies, such as orthogonal frequency division multiplexing (OFDM) [47,48] and non-orthogonal multiple access (NOMA) [49,50,51,52] can also be utilized to further enhance the system capacity, as well as offering flexible data rate allocation to multiple users for the optical interconnects. OFDM technology effectively uses bandwidth to counteract frequency-selective fading. However, in the era of exponential growth in communication data, spectrum resources have become increasingly scarce. NOMA technology, which supports large-scale data connections through non-orthogonal resource allocation, has been proposed to address this issue. This technology can also be used in optical interconnects or on-chip systems to provide flexible data allocation while supporting high data rate transmission.

In recent TOR application-specific-integrated-circuit (ASIC), 12.8 Tbit/s bandwidth is used by connecting 128 lanes of 100 Gbit/s QSFP-28 optical module [5]. The bandwidth density of QSFP-28 (data rate per module input/output width) is about 5 Gbit/s/mm [5]. In the advanced co-packaged optics (CPO) packaging, ASIC operating at 25.6 Tbit/s having dimensions of 90 mm × 90 mm is used. This means a 6.4 Tbit/s data rate should be carried out at each of the four sides in the CPO packaging [5]. Therefore, an ultra-high bandwidth density of 71 Gbit/s/mm should be needed. Technologies using parallel optics (i.e., spatial division multiplexing, SDM) or WDM alone may not meet this bandwidth density demand; hence, MDM is considered a promising candidate.

This work demonstrates a SiPh-based MDM optical power splitter that supports transverse-electric (TE) single-mode, dual-mode, and triple-mode (i.e., TE_0_, TE_1_, and TE_2_). In optical interconnects, an optical power splitter is needed for optical signal distribution and routing. However, a traditional optical splitter only divides the power of the input optical signal. This means the same data information are received at all the output ports of the optical splitter. The powers at different output ports may change depending on the splitting ratio of the optical splitter. The main contributions of our proposed optical splitter are: (i) Different data information are received at different output ports of the optical splitter via the utilization of NOMA. By adjusting the power ratios of different channels in the digital domain (i.e., via software control) at the Tx, different channel data information can be received at different output ports of the splitter. This can increase the flexibility of optical signal distribution and routing. (ii) Besides, the proposed optical splitter can support the fundamental TE_0_ mode and the higher modes TE_1_, TE_2_, etc. Supporting mode-division multiplexing and multi-mode operation are important for future optical interconnects since the number of port counts is limited by the chip size. This can significantly increase the capacity besides the WDM and spatial division multiplexing (SDM). The integrated SiPh MDM optical power splitter consists of a mode up-conversion section implemented by ADCs and a Y-branch structure for MDM power distribution. In the design of the MDM optical power splitter, although conventional empirical approaches can optimize the design parameters by scanning all possible configurations, it is time-consuming. Therefore, heuristic optical inverse design optimization methods have been used for geometric parameter retrieval targeting specific performance objectives. Similar evolutionary algorithms include evolution strategies (ES), genetic programming (GP), particle swarm optimization (PSO), etc. Among these, genetic algorithms (GA) can be used [53]. Through multiple iterations, the parameter set evolves to produce the optimal individual. Finally, to provide adjustable data rates at different output ports after the MDM optical power splitter, NOMA-OFDM is also employed. As a result, the proposed MDM optical power splitter with NOMA-OFDM can provide flexible data rate allocation for multiple users in optical interconnects and system-on-chip networks.

## 2. Structure Optimization by GA and SiPh-Based MDM Optical Power Splitter

GA optimization [45] is based on the principles of biological evolution, namely “survival of the fittest and elimination of the unfit”. During this process, genes are passed down to offspring through replication, resulting in minor variations. The offspring then pass on the mutated genes to the next generation in the same manner. This cyclic process continues, driving evolution. Moreover, in the design of SiPh devices, there are numerous degrees of freedom. We can exploit this characteristic by utilizing GA to optimize the devices and achieve optimal performance. GA can optimize several device parameters simultaneously. In this case, we need the mode theory to find out the approximate parameters. Then, instead of using the parameter optimization sweeping process, we use GA to enhance the efficiency of parameter search and optimization.

**Figure 2:**
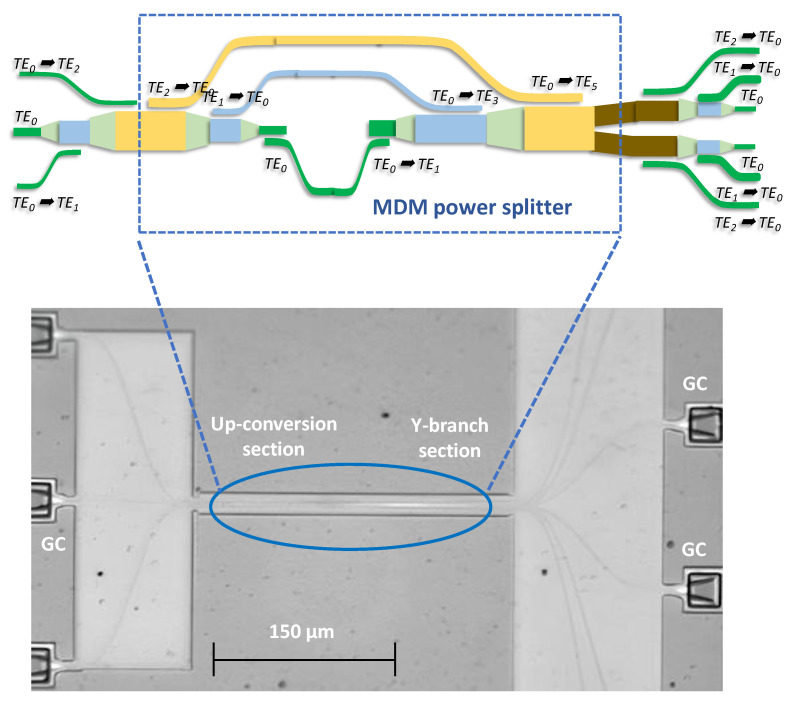
Schematic structure of the proposed SiPh MDM optical power splitter. Inset: photo of the MDM optical power splitter.

Figure 2 shows the MDM optical power splitter (i.e., inside the blue dotted box), which can be divided into two parts. The first part is an up-conversion section using ADCs to convert original TE_0_ to TE_1_, TE_1_ to TE_3_; and TE_2_ to TE_5_, respectively. The second part can equally power split the TE_1_ into 2 TE_0_, TE_3_ to 2 TE_1_, and TE_5_ to 2 TE_2_ via a Y-branch structure. Through GA optimization, the optimal device parameters for the MDM power splitter can be obtained. Here, the silicon access waveguide has dimensions of 0.35 μm × 0.22 μm, supporting the TE_0_ mode. The buried oxide (BOX) layer has a thickness of 2 μm, and the gap between ADCs in the coupling region is 0.15 μm. While satisfying the phase-matching condition, the bus waveguide widths for supporting the TE_1_, TE_3_, and TE_5_ are 0.74 μm, 1.525 μm, and 2.625 μm, respectively. The waveguide width of the Y-branch structure is 1.237 μm, enabling even power distribution among the TE_0_, TE_1_, and TE_2_ modes. For the measurement consideration using single-mode optical fiber (SMF) connecting to different equipment, the equal power split TE_2_ and TE_1_ modes should be converted back to TE_0_ mode via ADC, as shown in Figure 2. The TE_0_ mode signal in the silicon waveguide will be coupled in/out to the SMF via an on-chip gating coupler (GC). As shown in Figure 2, there are 3 GC inputs on the left-hand side and 6 GC outputs on the right-hand side. On the left-hand side, the optical signal launching at the center GC will preserve at the TE_0_ mode, while signals launching at the bottom and top GCs will be converted to TE_1_ and TE_2_ modes via ADCs, respectively. On the right-hand side, the 1st and 6th paths are for the power split TE_2_ mode, the 2nd and 5th paths are for the power split TE_1_ mode, and the 3rd and 4th paths are for the power split TE_2_ mode. Inset of Figure 2 shows the photo of the SiPh-based MDM optical power splitter fabricated by IMEC^®^.

**Figure 3:**
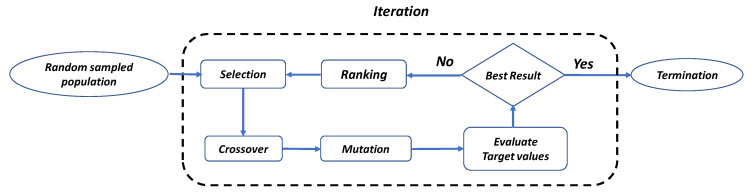
Flow diagram of the GA optimization algorithm.

Figure 3 illustrates the flowchart of the GA optimization process. Firstly, initial parameters are randomly selected as parents, with the coupling length, bus waveguide width, and access waveguide width parameters in the ADC serving as the genes of the parents. All genes are allocated within the same chromosome. To achieve the optimization objective of the structure, we utilize the built-in Python API in Lumerical^®^ for optimization. The optimized parameters are then used in device simulations using the Lumerical^®^ FDTD method, with forward transmission as the evaluation metric. This metric is defined by Equation (1),

(1) $$T_{forward}=\frac{{\left|a_{out}\right|}^{2}N_{out}}{{\left|a_{in}\right|}^{2}N_{in}}$$

where a is the arbitrary complex transmission coefficients for selected modes in the waveguide, N is the power contained in the mode selected in the waveguide. Here, ${\left|a_{in}\right|}^{2}N_{in}$ is the light source power set, ${\left|a_{out}\right|}^{2}N_{out}$ is the actual forward transmit power carried in the selected mode waveguide.

Next, we randomly select two individuals from the parents for crossover reproduction. Initially, the selection of parents is random, but subsequently, the parents are chosen based on their higher fitness scores. In the reproductive process, crossover points are selected within the parental genes, and offspring are generated by exchanging genes up to the crossover point positions. The newly generated offspring from crossover are then subjected to mutation. Based on a Gaussian distribution, random values are added to mutate the values in the genes, resulting in new mutation values. This is done to maintain diversity within the population and prevent premature convergence. After mutation, each offspring individual is evaluated to determine if convergence is achieved. In GA algorithms, this can be done using either an elitist GA algorithm or a non-elitist GA algorithm. In an elitist GA algorithm, several offspring are generated from each pair of parents.

**Figure 4:**
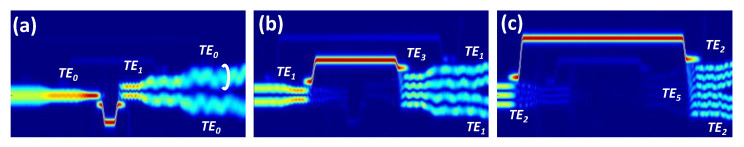
FDTD simulation results of the MDM optical power splitter at (**a**) TE_0_, (**b**) TE_1_, (**c**) TE_2_.

All individuals, including parents and offspring, are evaluated and ranked based on their fitness scores, and individuals with low fitness scores are eliminated. In contrast, in a non-elitist GA algorithm, two offspring are generated from each pair of parents, and all parents are eliminated. In our case, we use an elitist GA algorithm, where individuals with high fitness are reproduced through multiple iterations until the offspring show no significant differences compared to the previous generation (convergence), and the program terminates. If convergence is not reached, the surviving individuals will become the parent population in the next iteration, simulating the evolution of genes during mating. Figure 4a–c shows the Lumerical^®^ finite-difference time-domain (FDTD) simulation results of the MDM optical power splitter at TE_0_, TE_1_, and TE_2_ modes, respectively. For example, if the TE_0_ mode is required to be power split, it will be mode up-converted to the TE_1_ mode before power splitting. Similarly, the TE_1_ and TE_2_ are modes up-converted to the TE_3_ and the TE_5_ modes, respectively.

**Figure 5:**
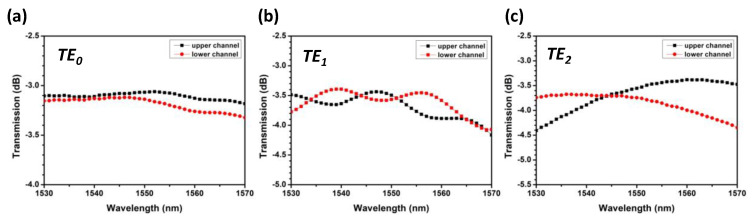
Losses and splitting ratios for the (**a**) TE_0_, (**b**) TE_1_, and (**c**) TE_2_ modes.

**Figure 6:**
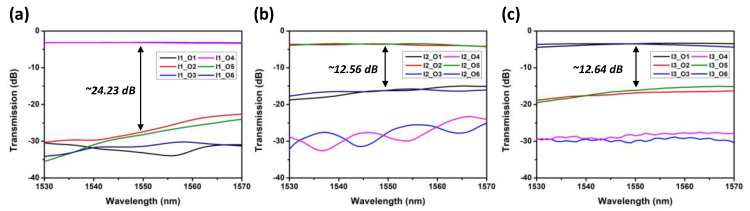
Crosstalk for the (**a**) TE_0_, (**b**) TE_1_, and (**c**) TE_2_ modes.

We also characterize the SiPh MDM optical power splitter at each mode. Figure 5a–c show losses and splitting ratios for the TE_0_, TE_1_, and TE_2_ modes. By excluding the intrinsic 3 dB loss in the power splitter, we can observe that the losses for the TE_0_, TE_1_, and TE_2_ modes are within ~0.5 dB, ~1 dB and ~1.5 dB, respectively. Figure 6a–c shows the crosstalk of power split TE_0_, TE_1_, and TE_2_ modes. Their crosstalk is ~24.23 dB, ~12.56 dB, and ~12.64 dB, respectively.

## 3. NOMA Algorithm for Flexible Allocation of Data

**Figure 7:**
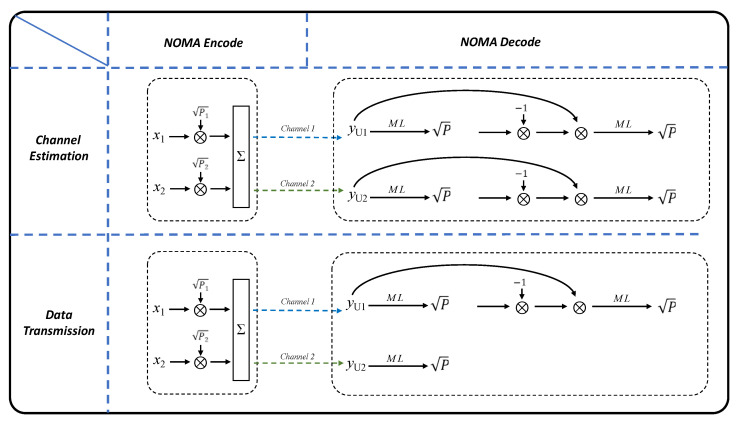
Flow diagram of the NOMA algorithm with channel estimation and data transmission.

The principle of NOMA is based on superposition coding and successive interference cancellation (SIC). At the transmitter (Tx) side, superposition coding is performed to overlay different power levels of data sequences. At the receiver (Rx) side, SIC is employed to perform subtraction decode of the data sequences. In the case of perfect decoding, the desired signals can be iteratively identified. Figure 7 illustrates the flowchart of the NOMA algorithm, which consists of channel estimation and data transmission.

The purpose of channel estimation is to obtain the channel state information (CSI), while data transmission aims to improve the data transmission rate and determine the decoding order. In the channel estimation stage, as shown in the upper part of Figure 7, the transmitted data is generated by adding two independent normalized power–quadrature amplitude modulation (QAM) data sequences to assist in determining the decoding order. Additionally, to enhance the data rate, the signal-to-noise ratio (SNR) distribution is also estimated. Since both users undergo two rounds of decoding, four SNR distributions are obtained $SNR_{U1}^{1st}$, $SNR_{U1}^{2nd}$, $SNR_{U2}^{1st}$ and $SNR_{U2}^{2nd}$. In this experiment, data-1 utilizes a lower power P_1_, while data-2 utilizes a higher power P_2_, as the channel coefficient (h_1_) for channel 1 is better than the channel coefficient (h_2_) for channel 2. Due to data-2 having greater coding capacity and data-1 being treated as noise, both user-1 (U_1_) and user-2 (i.e., U_2_) initially prioritize data-2 and employ maximum likelihood (ML) detection.

The entire channel estimation and SIC part can be represented by Equations (2) to (9),

(2) $$\sqrt{P_{2}}{\hat{x}}_{2,U1}\left(i\right)=\arg\;\underset{m}{max}\;f(y_{U1}\left(i\right)|\sqrt{P_{2}}x_{2,m})$$

(3) $$\sqrt{P_{2}}{\hat{x}}_{2,U2}\left(i\right)=\arg\;\underset{m}{max}\;f(y_{U2}\left(i\right)|\sqrt{P_{2}}x_{2,m})$$

where ${\hat{x}}_{2,U1}\left(i\right)$ and ${\hat{x}}_{2,U2}\left(i\right)$ are the *i*-th estimated symbols of the data-2 for U_1_ and U_2_, respectively. $y_{U1}\left(i\right)$ and $y_{U2}\left(i\right)$ are the *i*-th received signals of U_1_ and U_2_ after one-tap equalization, respectively. $f\left(x\right)$ is the Gaussian probability density function. $\sqrt{P_{2}}x_{2,m}$ is the set of normalized 4-QAM scaled with $\sqrt{P_{2}}$, here *m* = 1, 2, 3, 4.

Therefore, the first decoded *U*_1_ and *U*_2_ SNR distribution estimates can be expressed as Equations (3) and (4),

(4) $$SNR_{U1}^{1st}=\frac{1}{N}\sum_{i}\frac{|\;\sqrt{P_{2}}x_{2}\left(i\right)\;{|}^{2}}{|\;y_{U1}\left(i\right)-\sqrt{P_{2}}x_{2}\left(i\right)\;{|}^{2}}$$

(5) $$SNR_{U2}^{1st}=\frac{1}{N}\sum_{i}\frac{|\;\sqrt{P_{2}}x_{2}\left(i\right)\;{|}^{2}}{|\;y_{U2}\left(i\right)-\sqrt{P_{2}}x_{2}\left(i\right)\;{|}^{2}}$$

where $\sqrt{P_{2}}x_{2}\left(i\right)$ is *i*-th symbol of the data-2 scaled with $\sqrt{P_{2}}$ and *N* is the data length of the data-2.

Next, the received signals of *U*_1_ and *U*_2_ will be subtracted with the estimated data-2 sequence. It can be expressed as Equations (5) and (6).

(6) $$\sqrt{P_{1}}{\hat{x}}_{1,U2}\left(i\right)=\arg\;\underset{m}{max}\;f((y_{U1}\left(i\right)-\sqrt{P_{2}}{\hat{x}}_{2,U1}\left(i\right))|\sqrt{P_{1}}x_{1,m})$$

(7) $$\sqrt{P_{1}}{\hat{x}}_{1,U2}\left(i\right)=\arg\;\underset{m}{max}\;f((y_{U2}\left(i\right)-\sqrt{P_{2}}{\hat{x}}_{2,U2}\left(i\right))|\sqrt{P_{1}}x_{1,m})$$

Therefore, the second decoded *U*_1_ and *U*_2_ SNR distribution estimates can be expressed as Equations (7) and (8).

(8) $$SNR_{U1}^{2nd}=\frac{1}{N}\sum_{i}\frac{|\sqrt{P_{1}}x_{1}\left(i\right){|}^{2}}{|y_{U1}\left(i\right)-\sqrt{P_{2}}{\hat{x}}_{2,U1}\left(i\right)-\sqrt{P_{1}}x_{1}\left(i\right){|}^{2}}$$

(9) $$SNR_{U2}^{2nd}=\frac{1}{N}\sum_{i}\frac{|\sqrt{P_{1}}x_{1}\left(i\right){|}^{2}}{|y_{U2}\left(i\right)-\sqrt{P_{2}}{\hat{x}}_{2,U2}\left(i\right)-\sqrt{P_{1}}x_{1}\left(i\right){|}^{2}}$$

where $\sqrt{P_{1}}x_{1}\left(i\right)$ is *i*-th symbol of the data-1 scaled with $\sqrt{P_{1}}$.

In the data transmission stage, taking this experiment as an example, as shown in the lower part of Figure 7, the NOMA encoding and decoding process is performed with channel coefficients h_1_ being better than h_2_. At the Tx side, the power ratio (PR = $\sqrt{P_{2}/P_{1}}$) remains the same as in the channel estimation stage. Generally, the decoding order prioritizes the poorer channel coefficient and performs decoding only once, using higher power for encoding. Conversely, the better channel is encoded with lower power and undergoes multiple SIC iterations to improve the data transmission rate. Additionally, bit loading is applied based on the SNR distribution obtained from the channel estimation stage. In the encoding part, bit-loaded data sequences are generated for data-1 and data-2 based on the $SNR_{U1}^{2nd}$ and $SNR_{U2}^{1st}$ distributions, respectively, and encoded with $\sqrt{P_{1}}$ and $\sqrt{P_{2}}$), respectively. In the decoding part, the data sequences can be described mathematically, and their expressions are the same as Equations (2), (3) and (6). It is worth noting that if h_2_ is better than h_1_, in the encoding part, $\sqrt{P_{1}}$ will be replaced by $\sqrt{P_{2}}$, and $\sqrt{P_{2}}$ will be replaced by $\sqrt{P_{1}}$. In the decoding part, the expression of the data sequence will be similar to Equations (2), (3) and (7), but ${\hat{x}}_{1}$ becomes ${\hat{x}}_{2}$, and $x_{1}$ becomes $x_{2}$, and vice versa. By adjusting the PR for different users, NOMA can provide flexible data rate allocation for multiple users.

## 4. Experiment, Results and Discussion

**Figure 8:**
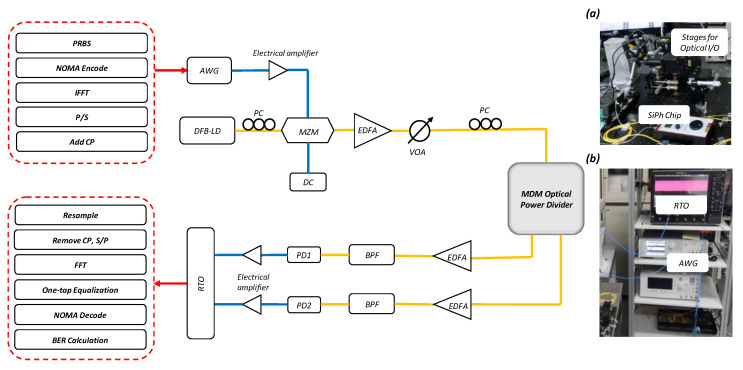
Experimental setup of the proposed MDM optical power splitter with NOMA-OFDM for flexible data rate allocation to users. AWG: arbitrary waveform generator; DFB-LD: distributed feedback laser diode; MZM: Mach-Zehnder modulator; PD: photodiode; RTO: real-time oscilloscope. Insets: Photos of (**a**) stages and SiPh chip, (**b**) AWG and RTO used the experiment.

Figure 8 illustrates the experiment of the proposed GA-optimized SiPh-based MDM power splitter with NOMA-OFDM for adjustable data rate allocation. The NOMA signal is generated by an arbitrary waveform generator (AWG, Tektronix^®^ AWG 70001). We use NOMA-OFDM signals with 170 subcarriers, an FFT size of 512, and a cyclic prefix (CP) length of 16 for modulation by combining two independent data sequences. Subsequently, OFDM encoding is performed, including inverse fast Fourier transform (IFFT), parallel-to-serial (P/S) conversion, and adding a cyclic prefix (CP). The electrical NOMA-OFDM signal is applied to a Mach-Zehnder modulator (MZM) with a bandwidth of 40 GHz to modulate the 1550.12 nm (ITU-34) wavelength optical signal emitted by a distributed feedback laser diode (DFB-LD). An erbium-doped fiber amplifier (EDFA) compensates for chip insertion loss. An optical bandpass filter (BPF) is employed to remove out-of-band amplified spontaneous emission (ASE) from the EDFA. The optical signal is coupled in and out of the proposed SiPh MDM power distributor using on-chip grating couplers (GC). During NOMA operation, the Rx simultaneously receives two output signals. Finally, the optical signal is simultaneously received by a 40 GHz photodiode (PD), and the NOMA-OFDM signal is captured by an 80 GS/s real-time oscilloscope (RTO, LeCroy^®^ 816ZI-B). The demodulation steps include resampling, CP removal, S/P conversion, FFT, one-tap equalization, NOMA decoding algorithm, and bit-error ratio (BER) calculation. Insets of Figure 8 show the photos of the experiment, illustrating the translation stages for holding the SiPh chip and the optical fibers for optical input/output (I/O); as well as the AWG and RTO for generating and detecting the NOMA-OFDM signal.

**Figure 9:**
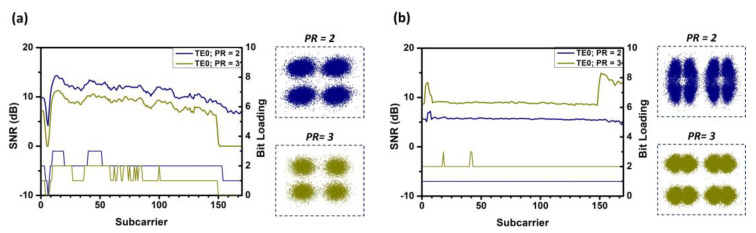
The SNR distribution, corresponding bit loading, and constellation diagram at different power ratios PR = 2.0 and PR = 3.0 at TE_0_, (**a**) user-1 and (**b**) user-2.

**Figure 10:**
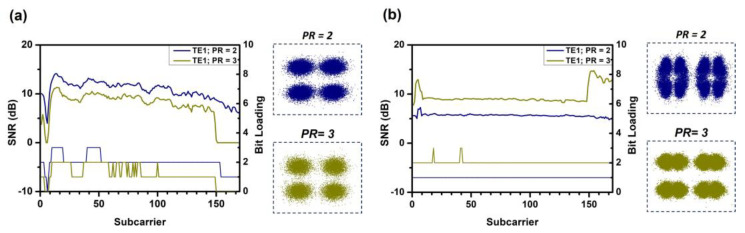
The SNR distribution, corresponding bit loading, and constellation diagram at different power ratios PR = 2.0 and PR = 3.0 at TE_1_, (**a**) user-1 and (**b**) user-2.

**Figure 11:**
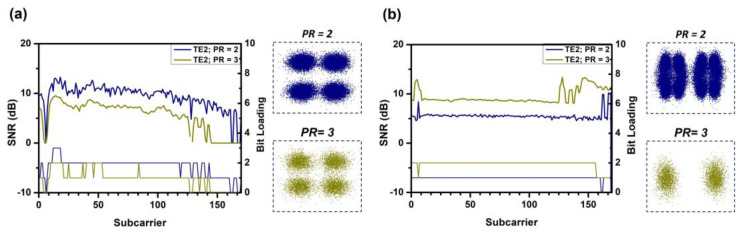
The SNR distribution and corresponding bit loading and constellation diagram at different power ratios PR = 2.0 and PR = 3.0 at TE_2_, (**a**) user-1 and (**b**) user-2.

Since the purpose of this concept demonstration is to validate the flexibility and performance of the system in transmitting data rates to different users flexibly in different modes, different PR ratios are used. In the experiment, we arbitrarily choose PR values of 2.0 and 3.0 for verification and evaluate the TE_0_, TE_1_, and TE_2_ modal channels. In the experiment, the optical power after the polarization controller is fixed at 11.1 dBm. For TE_0_ mode, the measured powers at the positions of user-1 and user-2 are −6.3 dBm and −7.4 dBm, respectively. For TE_1_ mode, the powers are −9.4 dBm and −10.04 dBm, respectively. For TE_2_ mode, the powers are −17 dBm and −20 dBm, respectively. Figure 9a,b show the SNR distribution and bit loading for user-1 and user-2 under the power allocation of TE_0_ mode. The blue curve represents PR = 2, and the green curve represents PR = 3. The corresponding constellation diagrams are provided in the insets of the figures. Since user-1 has a higher power, it undergoes two decoding processes, and the data sequence is encoded using P1. The SNR distribution decreases from PR = 2.0 to PR = 3.0. This can be explained by the increase in SNR for user-2. Figure 10a,b and Figure 11a,b show the SNR distribution and bit loading for user-1 and user-2 under the power allocation of TE_1_ and TE_2_ modes, respectively. They exhibit similar notches as observed in the power allocation of TE_0_ mode. It is worth noting that there is an abnormal dip near the fourth subcarrier caused by our electrical amplifier.

**Figure 12:**
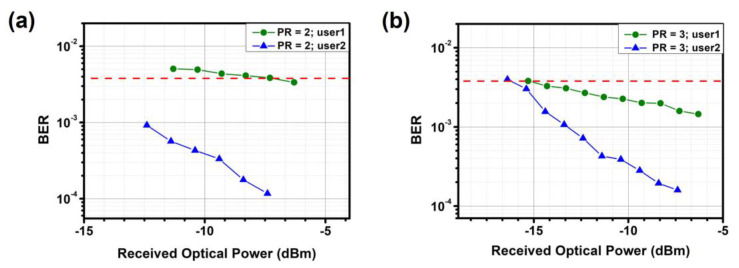
Measured BER curves for different users and power ratios at TE_0_ (**a**) PR = 2 (**b**) PR = 3. Red dotted line: HD-FEC threshold.

**Figure 13:**
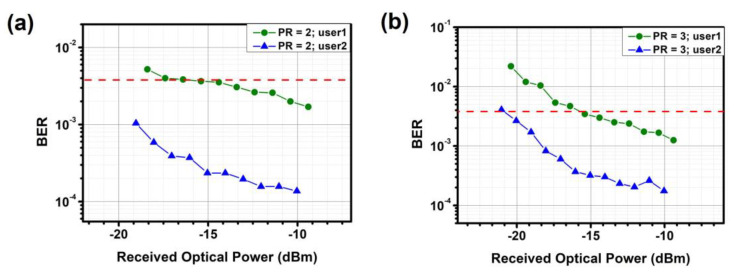
Measured BER curves for different users and power ratios at TE_1_ (**a**) PR = 2 (**b**) PR = 3. Red dotted line: HD-FEC threshold.

**Figure 14:**
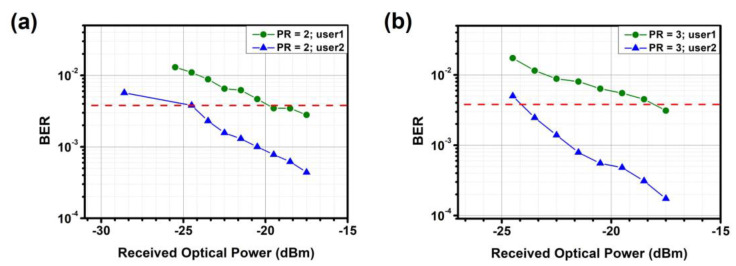
Measured BER curves for different users and power ratios at TE_2_ (**a**) PR = 2 (**b**) PR = 3. Red dotted line: HD-FEC threshold.

In addition, BER measurements are conducted on the proposed NOMA-OFDM-based MDM optical power allocator. Figure 12, Figure 13 and Figure 14 show the measured BER performance of user-1 and user-2 after optical power allocation in TE_0_, TE_1_, and TE_2_ modes, respectively. For TE_0_ and TE_1_, the data rates of user-1 and user-2 are 25.76 Gbit/s and 12.8 Gbit/s, respectively, when PR = 2.0. When PR = 3.0, the data rates are 15.08 Gbit/s and 26 Gbit/s, respectively. For TE_2_, the data rates of user-1 and user-2 are 22.65 Gbit/s and 12.65 Gbit/s, respectively, when PR = 2.0. When PR = 3.0, the data rates are 12.12 Gbit/s and 24.6 Gbit/s, respectively. Experimental results demonstrate that each measured channel meets the hard-decision forward-error-correction (HD-FEC, i.e., BER = 3.8 × 10^−3^) threshold.

## 5. Conclusions

Thousands of high-performance servers are interconnected inside data centers. The data center performance is highly affected by the performances of these interconnects and switches. Optical interconnects have gained much attention recently as a promising solution to provide high bandwidth, high throughput, low latency, as well as low power consumption when compared with electronic switches. Here, we proposed and demonstrated a SiPh-based MDM optical power splitter that supported single-mode, dual-mode, and triple-mode (i.e., TE_0_, TE_1_, and TE_2_). The optical power splitting and adjustable data rate allocation may be needed in future optical interconnects. The integrated SiPh MDM optical power splitter consisted of a mode up-conversion section implemented by ADCs and a Y-branch structure for MDM power distribution. The SiPh MDM optical power splitter was optimized by GA. By iteratively reproducing based on the “survival of the fittest” characteristic of GA, the time cost was reduced, and the optimal MDM power splitter parameters were optimized. Finally, to provide adjustable data rates at different output ports after the MDM optical power splitter, NOMA-OFDM was also employed. Furthermore, NOMA utilized channel differences to allocate the same resources for flexible data rate allocation. Experimental results showed that user-1 and user-2 can achieve data rate pairs of (user-1: 25.76 Gbit/s; user-2: 12.8 Gbit/s) and (user-1: 15.08 Gbit/s; user-2: 26 Gbit/s) when supporting higher-order mode conversion and meeting HD-FEC in TE_0_ and TE_1_ channels. In the TE_2_ channel, data rate pairs of (user-1: 22.65 Gbit/s; user-2: 12.65 Gbit/s) and (user-1: 12.12 Gbit/s; user-2: 24.6 Gbit/s) are achieved when PR = 2.0 and 3.0, respectively. Additionally, each channel can meet the requirements of HD-FEC. As a result, the proposed MDM optical power splitter with NOMA-OFDM can provide flexible data rate allocation for multiple users in optical interconnects and system-on-chip networks.

## Data Availability

The data presented in this study are available from the first author upon request.
